# A Focus on Intermediate-Risk Acute Myeloid Leukemia: Sub-Classification Updates and Therapeutic Challenges

**DOI:** 10.3390/cancers14174166

**Published:** 2022-08-28

**Authors:** Hassan Awada, Moaath K. Mustafa Ali, Bicky Thapa, Hussein Awada, Leroy Seymour, Louisa Liu, Carmelo Gurnari, Ashwin Kishtagari, Eunice Wang, Maria R. Baer

**Affiliations:** 1 Roswell Park Comprehensive Cancer Center, Buffalo, NY 14203, USA; 2 University of Maryland Greenebaum Comprehensive Cancer Center, Baltimore, MD 21201, USA; 3 Leukemia Division, Taussig Cancer Institute, Cleveland Clinic, Cleveland, OH 44106, USA; 4 Division of Hematology/Oncology, Department of Medicine, Medical College of Wisconsin, Milwaukee, WI 53226, USA; 5 Department of Translational Hematology and Oncology Research, Taussig Cancer Institute, Cleveland Clinic, Cleveland, OH 44106, USA; 6 University of California, Riverside, CA 92521, USA; 7 Department of Biomedicine and Prevention, University of Rome Tor Vergata, 00133 Rome, Italy; 8 Vanderbilt University, Ingram Cancer Center, Nashville, TN 37232, USA

**Keywords:** acute myeloid leukemia, intermediate-risk category, rational therapeutic strategies and challenges

## Abstract

**Simple Summary:**

Risk stratification models, including the European LeukemiaNet 2017 and 2022 guidelines, categorize newly diagnosed acute myeloid leukemia (AML) patients into several subgroups of distinct genetic characteristics and disease outcomes. The intermediate-risk group remains the most heterogenous group, as most AML patients fall into it (i.e., a basket category) by virtue of not fulfilling criteria that identify specific entities (e.g., core-binding factor AML, *TP53* mutations, complex karyotypes) of well-recognized prognostic significance. In this review, we aim to discuss the latest updates on intermediate-risk definition and highlight the therapeutic advances and challenges that warrant refining the prognostic classification of this category.

**Abstract:**

Acute myeloid leukemia (AML) represents a heterogeneous group of hematopoietic neoplasms deriving from the abnormal proliferation of myeloid progenitors in the bone marrow. Patients with AML may have highly variable outcomes, which are generally dictated by individual clinical and genomic characteristics. As such, the European LeukemiaNet 2017 and 2022 guidelines categorize newly diagnosed AML into favorable-, intermediate-, and adverse-risk groups, based on their molecular and cytogenetic profiles. Nevertheless, the intermediate-risk category remains poorly defined, as many patients fall into this group as a result of their exclusion from the other two. Moreover, further genomic data with potential prognostic and therapeutic influences continue to emerge, though they are yet to be integrated into the diagnostic and prognostic models of AML. This review highlights the latest therapeutic advances and challenges that warrant refining the prognostic classification of intermediate-risk AML.

## 1. Introduction

Acute myeloid leukemia (AML) is a diverse group of hematopoietic clonal disorders characterized by the accumulation of immature myeloid progenitors [1]. Despite recent advances in genomics and therapeutics, long-term outcomes are dismal in many AML subsets. Patient characteristics such as age and performance status (overall, summarized by the concept of fitness [2]) and heterogeneous cytogenetic and genomic underpinnings constitute the main determinants of outcomes [3]. Recent advances in genome scanning techniques have increased our understanding of AML pathogenesis, unveiling a plethora of driver gene lesions and possible disease-outcome modifiers [4]. Despite the abundance of genomic information on AML, current diagnostic and prognostic classifications somehow remain inexact. This is particularly true for the subgroup of patients classified as intermediate-risk according to the standard prognostic criteria used for AML clinical management (the European LeukemiaNet, ELN 2017 [5] and 2022 revision [6]). Apart from *FLT3* mutants, most patients usually fall into this group (an umbrella category) by virtue of not fulfilling criteria that identify specific entities (e.g., core-binding factor AML, *TP53* mutations, complex karyotypes) with well-recognized prognostic significance.

## 2. Intermediate-Risk Definition and Prognosis

AML classification and prognostic criteria are based on cytogenetic and molecular features at the time of diagnosis. At least 50% of cases exhibit karyotypic abnormalities by conventional cytogenetic analysis; in addition, mutational screening reveals at least one somatic alteration in virtually all patients [7,8]. Traditionally, AML has been categorized into favorable-, intermediate-, and adverse-risk groups [9]. While the favorable- and adverse-risk groups represent well-defined categories, the intermediate-risk group is generally characterized by the absence of favorable or unfavorable cytogenetic and molecular abnormalities and encompasses variable outcomes with standard-of-care therapies.

Modern genomic profiling using high-throughput technologies has led to the identification of molecular alterations reflective of disease biology and predictive of outcomes. For instance, identifying somatic mutations in the *FLT3*, *NPM1*, *CEBPA*, *IDH1*, *IDH2*, *DNMT3A*, *TET2*, and *KIT* genes provided important information that improved AML diagnosis, treatment stratification, and monitoring [10,11,12]. The ELN AML risk stratification in 2017 [5] (Table 1) integrated additional cytogenetic and molecular abnormalities [5] not present in the 2010 version. A subsequent study of 1116 AML patients validated the ELN 2017 prognostic stratification and showed that it assigned more patients to the favorable- and adverse-risk groups than the 2010 criteria, thereby shrinking the intermediate-risk group [13]. Nevertheless, this remained an ill-defined “basket category” for patients not fulfilling the criteria for the favorable- or adverse-risk groups. Moreover, with regard to clinical management, while current indications for consolidation treatment with allogeneic hematopoietic stem cell transplantation (HSCT) are well-established in favorable- and adverse-risk patients, information derived from disease monitoring is crucial in clinical decision-making in intermediate-risk patients because of their heterogeneity [14,15].

Interestingly, in a retrospective study of 863 de novo AML patients < 60 years, Eisfeld et al. found additional gene mutations able to refine the prognostic classification of ELN 2017 [16]. In this study, the authors found a total of 2354 mutations, averaging 3 per individual. *WT1/NPM1* co-mutant, *DNMT3A*, and *ZRSR2* mutated patients had very poor survival outcomes, similar to those of the adverse-risk group. Of note, *BCOR*- and *SETBP1*-mutated AML patients were categorized as favorable-risk, whereas *IDH* mutants were associated with adverse-risk patients and had outcomes similar to those of intermediate-risk patients. They also found that *FLT3* internal tandem duplication *(ITD)* with a high allelic ratio had survival outcomes similar to those of the adverse-risk group rather than the intermediate-risk category, regardless of *NPM1* mutational status. Based on these findings, 9% of favorable-risk, and 53% of intermediate-risk AML patients per ELN 2017 could be reclassified as belonging to the adverse-risk category, and 9% of adverse-risk and 4% of favorable-risk patients could be reclassified as intermediate-risk. Thus, the current AML prognostic criteria do not encompass the molecular heterogeneity inherent to the disease, and the inclusion of mutations in additional myeloid genes may refine classification [17]. However, identifying genomic alterations and their prognostic significance is still an evolving field, and guidelines will require ongoing refinements.

Given the expansion of the AML therapeutic arsenal, consideration of the mutational status of additional genes is of utmost importance also in older patients. Recently, the ALFA-1200 study evaluated genomic predictors of outcomes in 471 newly diagnosed AML patients ≥ 60 years treated with standard “7 + 3” induction chemotherapy [18]. The group developed a decision tool to predict survival outcomes in older AML patients using mutations in seven genes, *NPM1*, *NRAS*, *KRAS*, *ASXL1*, *DNMT3A*, *TP53*, and *FLT3-ITD*. In patients with poor-risk cytogenetics, *TP53* and *KRAS* mutations were independently associated with poor survival, whereas *SETBP1*, *NRAS*, *ASXL1*, and *RUNX1* status predicted lower complete remission rates even in patients with favorable- and intermediate-risk cytogenetics. Additionally, mutations in *DNMT3A*, *ASXL1*, *NRAS*, *NMP1*, and *FLT3-ITD* (regardless of allelic ratio) were independent predictors of overall survival (OS). When cytogenetic risk and mutations in the seven genes were combined, 39.1% of patients had a 2-year OS of 66.1% (assigned to “go-go” group), 7.6% of patients had a 2-year OS of 2.8% (assigned to “no-go” group), and 3.3% of patients had a 2-year OS of 39.1% (assigned to “slow-go” group). This genetic-based predictive model was validated in three independent cohorts.

**Figure 1:**
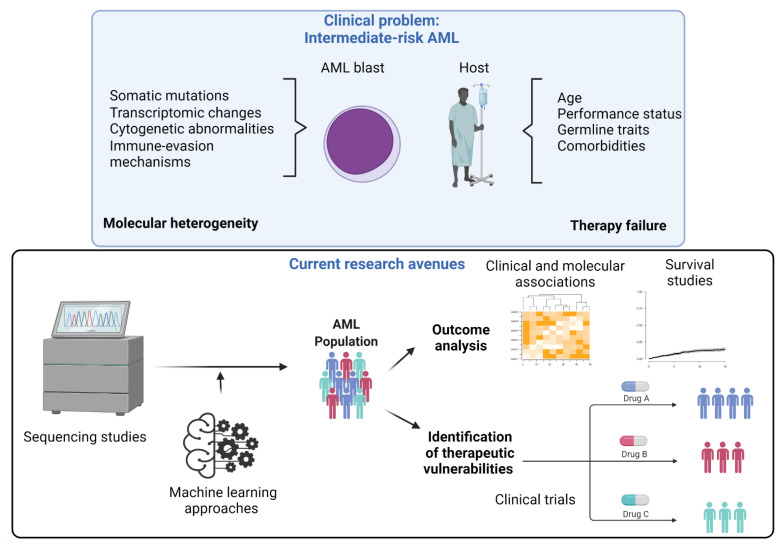
The clinical problem of intermediate-risk AML. The upper panel summarizes features responsible for molecular heterogeneity, and host-related outcome modifiers are summarized. In the lower panel, current research approaches to ameliorating the outcomes of such patients are illustrated. Specifically, the application of genome scanning techniques and machine learning methods may help better characterize AML populations to assess geno-phenotypic associations and identify therapeutic vulnerabilities.

Besides genomic characteristics at the time of diagnosis, host-related factors such as poor baseline functional status, multiple comorbidities, and advanced age are associated with poorer prognosis (Figure 1) [19].

Interestingly, machine learning (ML) techniques have been applied in leukemia research and represent a promising auxiliary tool for developing new approaches that aim to improve AML risk stratification. Several studies have implemented unsupervised and unbiased ML methods that demonstrated high accuracy in terms of genomic classification [20]. These approaches may be further employed to define ambiguous definitions by unmasking unexplored clinico-morphologic and genomic characteristics unique to intermediate-risk AML patients. Indeed, ML-based methods demonstrated a 97% accuracy rate when reclassifying AML patients according to four genomic signature clusters [4]. While the clusters exhibited a certain degree of overlap with the 2017 ELN groups, significant differences in survival across the two classification systems emphasized the need for a more subtle distinction of AML risk groups.

Most recently, the 2022 ELN guidelines for diagnosis and management of AML were published [6]. In this newest version, the risk classification of AML has changed to using a hierarchical model that depends on molecular aberrations detected rather than morphologic or historical criteria (e.g., therapy-related, secondary AML). Moreover, the intermediate-risk AML group was redefined with the abrogation of the value given to *FLT3* allelic ratio, as demonstrated in Table 2.

## 3. Current Therapeutic Approaches

The current AML treatment paradigm recommended by the major societies, including NCCN and ESMO, is heavily based on four factors: age, performance status, organ function (in other words, patients’ fitness), and risk classification per ELN 2017 criteria [5]. Of note is that there is a lack of trials solely focusing on patients with intermediate-risk AML. Hence, the current evidence-based therapeutic approaches in this population are mainly extrapolated from large studies that included different risk categories of patients. In this scenario, it is essential to recognize that the heterogeneity in the studied populations makes generalization difficult and might over- or underestimate the actual benefit of any treatment in such subgroup. In addition, none of the current Food and Drug Administration (FDA)-approved agents are specific for the intermediate-risk category, thereby configuring an unmet clinical need in AML. As the prognostication of AML continues to evolve and trial inclusion criteria change, therapeutic recommendations from major societies will likely be modified accordingly. Moreover, since the criteria used to define risk groups significantly vary between trials, it is challenging to determine the benefit of treatments and compare it among different studies.

Treatment of intermediate-risk ELN patients eligible for intensive therapy has not substantially changed over the past few decades, and “7 + 3” induction therapy still represents the standard of care. Data from three randomized trials comparing anthracycline doses (daunorubicin 45 mg/m^2^ and 90 mg/m^2^) show a survival benefit with higher doses of anthracyclines in both younger and older patients (at least up to the age of 65 years) [21,22,23]. These regimens lead to complete remission (CR) in 60–80% of younger adults and 40–60% of older patients. The benefit of adding gemtuzumab ozogamicin (GO), a humanized anti-CD33 monoclonal antibody conjugated to calicheamicin, to standard therapy was limited to favorable- and intermediate-risk patients [24,25,26].

Table 3 summarizes therapeutic regimens recommended by the 2021 NCCN and the 2020 ESMO guidelines for use in intermediate-risk AML. In this subgroup, for patients aged <60 years and with intact organ function, current recommendations support induction chemotherapy, as described above [27,28]. Patients who achieve a CR to induction chemotherapy receive consolidation chemotherapy, most commonly with high-dose cytarabine, but this may vary based on the chosen induction regimen. [29]. Cytarabine administered at 3 g/m^2^ (high-dose) by 3 h infusion, every 12 h on days 1, 3, and 5, was associated with a lower relapse rate and improved OS compared to intermediate- and low-dose regimens. However, there was no subgroup analysis according to cytogenetics in the original trial [29]. A follow-up study evaluated the outcomes of high-dose cytarabine in patients stratified into three cytogenetic groups, based on the presence of core-binding factor rearrangements, a normal karyotype, and other cytogenetic abnormalities. In the modern era, intermediate-risk AML would constitute parts of the two latter groups. The 5-year relapse-free survival with high-dose cytarabine was 78%, 40%, and 21% in these three groups, respectively, and was superior to results obtained with the intermediate- and low-dose cytarabine regimens (*p* < 0.05) [30].

Since most studies have focused on stratifying patients to various first-line therapies, little is known about the outcomes of different consolidation regimens in the intermediate-risk AML group. As mentioned, patients who remain in remission may be considered for HSCT as a potentially curative option, when suitable for the procedure. However, randomized trials investigating the benefit of transplant in intermediate-risk AML are lacking. Studies investigating the benefit of allogeneic and autologous HSCT have used donor availability as a criterion for transplant [31,32]; hence, it remains difficult to assess precisely the absolute benefit of this procedure in this group. Moreover, because the definition of the intermediate-risk AML group continues to change along with the advancements in risk stratification and measurable residual disease (MRD)-directed treatment strategies, determining the benefit of consolidative allogeneic HSCT in this population remains challenging and must account for individual patients’ characteristics (disease status, MRD, performance status, and donor availability). In a retrospective analysis, AML patients, less than 60 years old, who underwent allogeneic HSCT had a trend toward improved relapse-free survival (RFS), OS, and a lower relapse rate than their non-transplanted counterparts, but the results did not reach statistical significance [33]. HSCT is currently considered an option after remission induction in the intermediate-risk group, per NCCN and ESMO guidelines [27,28]. Nevertheless, we recommend that the treating physician discusses the role of such a procedure with a patient diagnosed with intermediate-risk AML, including the potential benefits and risks. While consolidation with allogeneic HSCT is a potential option in younger patients who are generally fit and less likely to suffer from treatment-related morbidity and mortality, enrollment in clinical trials may be a reasonable alternative in cases rendered not medically fit for HSCT.

The role of MRD in treatment stratification is also evolving. MRD refers to the detection of malignant cells by molecular biology or flow cytometry techniques in patients in complete remission, defined as hematologic recovery and an absence of AML blasts by morphological bone marrow examination. Studies have demonstrated the usefulness of monitoring MRD in patients with RUNX1-RUNX1T1 rearrangements and *NPM1* mutations [34,35]. Patients with these abnormalities that become undetectable by molecular techniques tend to have better OS and lower cumulative rates of relapse [36]. However, the application of MRD monitoring in clinical settings remains complicated, mainly due to conflicting results with other molecular abnormalities and different sensitivities inherent to the techniques used [37,38,39]. Other challenges include standardizing the approaches by which MRD is analyzed and interpreted, as well as determining the optimal time and specimen source for its measure [40]. Nevertheless, a growing body of evidence suggests a promising role for MRD monitoring in guiding therapy choices in intermediate-risk AML patients. For instance, in the Italian GIMEMA AML1310 trial, in which the NCCN 2009 recommendations were used for risk classification, intermediate-risk patients were allocated into post-consolidation autologous or allogeneic HSCT, in cases with MRD-negative or MRD-positive status using multiparametric flow cytometry (MFC), respectively [41]. No significant difference was seen in the 2-year OS (79% vs. 70%, *p* = 0.713) or disease-free survival (DFS) (61% vs. 67%, *p* = 0.773) rates between the MRD-negative and -positive groups. Remarkably, MRD-positive intermediate-risk AML patients treated with allogeneic HSCT had OS and DFS rates similar to favorable-risk patients. The same group confirmed the role of MRD when risk categorization according to the ELN 2017 recommendations was applied to the AML1310 trial [42]. Indeed, no significant difference in OS at two years was observed between intermediate-risk MRD-negative patients who received either autologous or allogeneic HSCT (85.7% vs. 77.8%, *p* = 0.234), whereas allogeneic HSCT did significantly increase survival compared to autologous HSCT in MRD-positive patients (75% vs. 0%, *p* = 0.0231).

Maintenance therapy is used to decrease the risk of disease relapse. In an open-label phase III study, patients ≥ 60 years with AML or refractory anemia with excess blasts who achieved remission post induction were randomized to receive azacitidine 50 mg/m^2^ subcutaneously days 1–5 every four weeks vs. observation. While patients treated with azacitidine had improved 12-month DFS (64% vs. 42%, *p* < 0.05), OS was similar (84% vs. 70% at 12 months, *p* = 0.69). Of note, the study design did not require patients to receive consolidation with cytarabine, and no subgroup analysis based on risk stratification was provided in this trial [43]. In another study of patients ≥ 55 years with de novo or secondary AML, with intermediate- or poor-risk cytogenetics according to 2011 NCCN risk classification, who were not candidates for HSCT, oral azacitidine 300 mg daily for 14 days per 28-day cycle improved OS compared to placebo (24.7 months and 14.8 months, respectively; *p* < 0.05) [44]. A subgroup analysis showed improved 2-year OS (difference 13.6%, 95% CI: 3.9–23.4) in the intermediate-risk group.

In patients who do not achieve remission with induction or relapse after treatment, the current NCCN and ESMO guidelines recommend targeted therapy or clinical trial enrollment whenever possible. Table 3 summarizes commonly used options in this setting. As with first-line regimens, most trials included intermediate-risk group AML in the study populations.

## 4. Clinical Trials

As discussed, meticulous clinical research from bench to bedside has led to a better understanding of the pathophysiology of AML and, subsequently, to the discovery of novel therapeutic agents. Nevertheless, the molecular heterogeneity of AML continues to remain a challenge. Indeed, the significant advances in elucidating the genomics of AML have not yet led to ameliorating outcomes, which remain dismal in relapsed and refractory patients (R/R AML). Moreover, the situation is even hazier when considering the treatment of older and frail (unfit) AML patients.

In the United States, more than 300 clinical trials are currently assessing the safety and efficacy of various therapeutic agents in AML patients. A detailed review of ongoing studies is beyond the scope of this review; however, in Table 4, we have listed some representative examples currently enrolling intermediate-risk AML patients who are newly diagnosed or relapsed/refractory.

## 5. Targeted Agents and Challenges in Intermediate-Risk AML

The entire schema of ELN risk classification currently used in the clinic is derived from the experience of patients treated with intensive chemotherapy. Over the past few years, with the advent of many new drugs for AML, including venetoclax combinations and a molecularly defined class of targeted therapies, *IDH1/2* or *FLT3* inhibitors, it is uncertain if this classification would still apply [59].

### 5.1. FLT3 Inhibitors

Mutations in the *FLT3* gene are among the most common mutations in intermediate-risk AML, most commonly associated with a normal karyotype [60]. These mutations lead to constitutive activation of the *FLT3* receptor tyrosine kinase and multiple downstream signaling cascades involved with AML cell survival, proliferation, and differentiation. In the ELN 2017, the allelic burden and the pattern of co-mutations (in particular in *NPM1* and *DNMT3A*) modulated the prognostic significance of the more common and prognostic *FLT3* mutation, *FLT3*-ITD [7,61]. For instance, *FLT3-ITD* with a high allelic ratio (>0.5) conferred adverse prognosis; however, the simultaneous presence of an *NPM1* mutation made it instead recognized as intermediate-risk [4]. The new 2022 revision abated this differentiation by assigning all *FLT3* mutants to the intermediate-risk category [6]. The treatment paradigm for *FLT3*-mutated AML patients has been transformed by the advent of small molecule tyrosine kinase inhibitors (midostaurin, gilteritinib) for both newly diagnosed and R/R AML [54,62]. Furthermore, the combination of azacitidine and venetoclax has changed treatment outcomes for adults who are not candidates for intensive induction chemotherapy [60,63]. Response rates of *FLT3*-mutated AML patients to the azacitidine/venetoclax combination are comparable to those of patients with wild-type *FLT3*. In light of response improvements from doublets, the need to study rational combinations of triplets or sequential therapies to continue improving the outcomes of patients with AML with *FLT3*-ITD is a current topic of research.

### 5.2. IDH1/2 Inhibitors

Characterization of the unique mechanism of leukemogenesis resulting from *IDH1* and *IDH2* mutations in AML led to the development of targeted oral therapies. This represents a remarkable bench-to-bedside journey. *IDH1/2* mutations are present in ~20% of AML cases (*IDH1*: ~5%–8% and *IDH2*: 9%–12% [61]). These mutations frequently occur in normal-karyotype AML, representing a common subset of intermediate-risk diseases. First-in-class, oral, selective inhibitors of mutant *IDH1* (ivosidenib) [64] and *IDH2* (enasidenib) [65] are FDA-approved for the management of R/R AML, based on phase 1 clinical trials showing overall response rates of 42% and 39%, respectively, along with manageable toxicity profiles. Despite the initial success, several questions were raised based on the preliminary results of the recent randomized trials. The phase 2 study comparing azacitidine with or without enasidenib as a first-line treatment for AML patients who are unfit for intensive chemotherapy showed improved response rates but similar OS as the combination therapy [66]. Similarly, the phase 3 IDHENTIFY study enrolling *IDH2*-mutant R/R AML patients (after the failure of two or three lines of therapy) reported no survival benefit with enasidenib compared with standard of care [67]. These two studies highlight the challenges in deciding the optimal approach to using targeted therapies in AML and the need for randomized studies. Besides, a recent phase 3 trial evaluating azacitidine with or without ivosidenib in *IDH1*-mutant newly diagnosed AML unfit for intensive treatment showed a 12-month event-free survival rate significantly higher in the experimental group, along with a benefit in OS (24.0 vs. 7.9 months; *p* = 0.001), opening new therapeutic opportunities in such a setting. [58] Particularly, the use of ivosidentib plus azacitidine enabled the clearance of detectable *IDH1*-mutated cells, and less transfusion support was needed, with safety signals similar in the two groups if excepting the higher rate of differentiation syndrome in the experimental arm.

### 5.3. Others

CA-4948 is a potent interleukin-1 receptor-associated kinase 4 (IRAK-4)/*FLT3* inhibitor, with potential for anti-tumor activity in AML, that is currently in the early phases of therapeutic development (NCT04278768). In adult patients with *FLT3*-mutated R/R AML, combinations with experimental oral agents such as TL-895 (tyrosine kinase inhibitor) and KRT-232 (MDM2 inhibitor) are being evaluated for safety and efficacy in an early phase clinical trial (NCT04669067). Minnelide is a potent heat shock protein 70 inhibitor that showed potent preclinical anti-tumor activity in AML [68]. A phase 1 study (NCT03760523) is ongoing to investigate this novel agent in patients with R/R-AML who are ineligible for intensive chemotherapy.

### 5.4. Challenges

Many challenges remain unsolved in intermediate-risk AML. A first challenge is to define the precise scenarios for using the plethora of novel targeted drugs [69]. The ELN criteria, including the latest revision, are based on data derived from intensively treated patients, thereby leaving unanswered the question if current schemes may still be valid in patients treated with newer agents [6]. This is particularly true for *FLT3*-positive cases, now representing a good fraction of the intermediate-risk group according to the latest classification, especially in light of the availability of specific targeted treatment options. A second challenge is represented by AML trial designs, which beg the question of what is the best setting to test these novel compounds. Indeed, since the intermediate-risk group is the most heterogeneous category, issues of clonal evolution and genomic and/or epigenomic complexity, after receiving standard of care therapies, and the emergence of subclones with distinct resistance mechanisms are only some of the challenges to be faced. A third challenge is the incorporation of novel agents and existing targeted therapies in the frontline regimens, which are likely to increase the proportion of patients achieving and maintaining CR for longer periods [70]. As a consequence, and with deeper responses resulting from the use of tailored therapeutic approaches in the induction schemes, the definition of the need for consolidation/maintenance regimens and HSCT will urge further refinements. While guiding treatment strategies by MRD-based approaches could answer some of these questions, individual patients’ characteristics and inherent subgroup genomic features (e.g., disease subclassifications exemplified in the recent WHO revision and other classifications [71,72]) may represent other difficult obstacles to overcome.

## 6. Immunotherapies in AML

Over the last decade, immunotherapies have shown encouraging anti-tumor activity across most cancer types and may offer a therapeutic opportunity also in intermediate-risk AML, in a way that is mutation-agnostic. Immune-based therapeutics, including monoclonal antibodies, immune checkpoint inhibitors (ICIs), bispecific T cell engagers (BiTE) antibodies, and chimeric antigen receptor T-cell therapy (CAR-T) are being extensively investigated to improve the outcome of AML patients.

### 6.1. The Role of Immune Checkpoint Inhibitors in AML

Immune checkpoints, namely cytotoxic T lymphocyte antigen 4 (CTLA-4), programmed death-1 (PD-1), and programmed death-ligand 1 (PD-L1), play a crucial role in regulating T cell immune response to the tumor cells [73]. ICIs, such as ipilimumab, nivolumab, pembrolizumab, and atezolizumab, have revolutionized the treatment landscape in various solid tumors. ICIs promote anti-tumor activity by enhancing T cell activation and preventing immune escape of tumor cells [74]. The role of ICIs in the management of AML is evolving, and various trials are ongoing to evaluate its efficacy. High expression of PD-1, PD-L1, and T-cell immunoglobulin mucin domain 3 (TIM3) and co-expression of PD-1/CTLA-4, PD-1/PD-L1, PD-1/LAG-3, and PD-L2/CTLA-4 are associated with poor prognosis in AML, particularly in patients with *FLT3*, *RUNX1*, and *TET2* mutations [75,76,77]. High PD-1, PD-L1, and PD-L2 expression could potentially contribute to resistance to treatment with azacytidine [76]. However, a recent randomized phase II clinical trial of Azacitidine in combination with PD-L1 inhibitor (Durvalumab) in older patients (≥65 years) with AML failed to show clinical improvement, compared to azacitidine alone [78]. Of note is that in this study, the response to treatment was not associated with PD-L1 expression, DNA methylation, and mutational status. In a phase I/Ib clinical trial, the efficacy, and safety of ipilimumab were evaluated in patients with relapsed hematologic malignancies after allogeneic hematopoietic stem-cell transplantation (Allo-HSCT) [79]. Out of 28 patients enrolled, 12 were relapsed AML, and no response was observed in patients who received 3 mg/kg of ipilimumab. However, dose escalation to 10mg/kg demonstrated anti-tumor activity; CR was noted in 5 out of 12 relapsed AML patients (4 with extramedullary leukemia and 1 with secondary AML), with a durable response of more than 1 year in 3 patients. In another phase I clinical trial, PD-1 blockade (Nivolumab) was evaluated in patients with hematologic malignancies after Allo-HSCT [80]. Out of 28 patients enrolled in the clinical trial, 10 were AML. Only minimal response was observed in AML patients, and immune-related adverse events such as graft vs. host disease occurred even with lower doses of Nivolumab.

Ravandi et al. evaluated nivolumab in combination with Idarubicin and cytarabine in newly diagnosed AML patients. In this single-arm, phase 2 clinical trial, 44 patients were enrolled, of whom 17 (39%) were intermediate-risk AML based on ELN 2017 classification [77]. The median overall survival was 18.54 months. Overall, 19 patients had a response and subsequently underwent Allo-HSCT. Of note, the authors reported higher CD4+ T-cell co-expression of PD-1/TIM3 and PD-1/LAG-3 in non-responders compared to responders.

### 6.2. Monoclonal Antibodies and CAR-T Cell Therapy in AML

CD33 is highly expressed on AML cells, and unconjugated and conjugated CD33 monoclonal antibodies are in the early phase of drug development [81]. Current clinical trials targeting CD33 with GO (NCT03672539, NCT04207190, NCT03839446) are outlined in Table 4. For instance, lintuzumab Ac225, a radiolabeled anti-CD33 antibody, combined with chemotherapy, demonstrated a clinically acceptable safety profile [82]. A phase I/II study (NCT03441048) is underway to assess the safety and efficacy of this novel therapeutic agent in R/R AML. Flotetuzumab is a humanized bispecific antibody-based molecule binding to CD3 and CD123. Results from a phase 1/2 study (NCT02152956) demonstrated acceptable safety and evidence of anti-tumor activity in R/R AML patients [83]. A phase 2 study is ongoing to evaluate its efficacy in AML that has relapsed following allogeneic HSCT (NCT04582864).

Another active area of experimental drug development is CAR-T cell therapy [84]. In AML, targets such as CD33, CD123, CLL1, and CD44v6 are the most frequent (NCT04789408, NCT04219163, NCT03190278). Notably, commonly expressed CAR-T targets are also present in normal myeloid cells, leading to potential myelosuppression [81,85]. Newer generations of CAR-T cell products could potentially overcome these limitations. For instance, CD70 was recently identified as a promising target for CAR-T cell therapy in AML because of its selective expression on both leukemic blasts and leukemic stem cells [85], providing a rationale for targeted therapy in AML without adversely affecting normal hematopoiesis [86]. In a recent study, two CD70-CAR T constructs exhibited significant anti-tumor efficacy in vitro and in vivo [87], effectively eliminating AML cells and sparing normal hematopoietic stem cells, thereby avoiding potentially dangerous on-target/off-tumor toxicity.

## 7. Conclusions

AML is a disorder characterized by a puzzled molecular landscape. As a result, patients are characterized by different outcomes according to their individual genomic makeup, which, together with host factors such as age and performance status, contribute to the diverse therapeutic and survival scenarios. Intermediate-risk cases, as per ELN 2017, and more recently ELN 2022, represent the most heterogeneous group because of the broad features included in the definition of this category (or, better yet, the handful of characteristics used for the inclusion in the other two groups). While new genomic information is generating a more detailed dissection of the disease, as in the recent two classifications of myeloid disorders [71,72], an urgent need for a redefinition of diagnostic and prognostic criteria is vital for both better trial designing and adequate indications for new targeted treatments. Immunotherapies also offer potential opportunities, but this area needs further refinements and additional basic and translational studies in AML in general. Furthermore, the application of modern machine learning approaches with the current availability of genomic information and big data platforms will potentially unravel specific subgroups of patients, further contributing to identifying discrete differences in AML patients, particularly in the intermediate-risk group.

Perhaps, as our understanding of AML pathogenesis and risk-stratification modalities continues to improve, we anticipate that the intermediate-risk group definition will continue to change. In fact, the exponential growth in the targeted therapeutic options utilized in AML continues to alter the prognostic significance of many molecular derangements. Nonetheless, in harmony with the 2022 ELN guidelines, we recommend judicious incorporation of the patient’s fitness for the intensive regimens and disease molecular characteristics used as criteria for treatment selection.

## Figures and Tables

**Table 1:** 2017 ELN intermediate-risk stratification by genetics. Adapted from [5].

Intermediate-risk category	Mutated *NPM1* and *FLT3*-ITD^high^ †
	Wild-type *NPM1* without *FLT3*-ITD or with *FLT3*-ITD^low^ † (without adverse-risk genetic lesions)
	t(9;11)(p21.3;q23.3); *MLLT3-KMT2A* ‡
	Cytogenetic abnormalities not classified as favorable or adverse

† Low, low allelic ratio (<0.5); high, high allelic ratio (≥0.5). ‡ The presence of t(9;11)(p21.3;q23.3) takes precedence over rare, concurrent adverse.

**Table 2:** 2022 European LeukemiaNet (ELN) risk classification by genetics at initial diagnosis. Adapted from [6].

Intermediate-risk category	Mutated *NPM1* †‡ with *FLT3*-ITD
	Wild-type *NPM1* with *FLT3*-ITD
	t(9;11)(p21.3;q23.3)/*MLLT3::KMT2A* *†
	Cytogenetic and/or molecular abnormalities not classified as favorable or adverse

† Mainly based on results observed in intensively treated patients. Initial risk assignment may change during the treatment course, based on the results from analyses of measurable residual disease. ‡ AML with NPM1 mutation and adverse-risk cytogenetic abnormalities are categorized as adverse-risk. * The presence of t(9;11)(p21.3;q23.3) takes precedence over rare, concurrent adverse-risk gene mutations.

**Table 3:** Major therapeutic regimens and associated trials suggested in NCCN guidelines for intermediate-risk AML.

Drug/Regimen	Trial/Year	AML-Specific FDA Approval	Design/Setting	Study Population	Experimental Arm	Comparison Regimen	Age Group and Characteristics	Risk Group	Pertinent Finding	Remarks
**Newly Diagnosed AML-Induction-Eligible**										
7 + 3 Regimen	Fernandez [23]/2009	Daunorubicin: remission induction in AML (myelogenous, monocytic, erythroid) in adults.	Multi-institutional, randomized, open-label trial	De novo or secondary AML.	Induction: daunorubicin 60 mg/m^2^ IV days 1–3 with Ara-C 100 mg/m^2^ continuous IV infusion days 1–7.	Induction: daunorubicin 45 mg/m^2^ IV days 1–3 with Ara-C 100 mg/m^2^ continuous IV infusion days 1–7.	17–60 years	No risk groups excluded	HR for death in the high-dose daunorubicin group 0.74 (*p* < 0.05). Improved OS (HR 0.8, *p* = 0.02) in intermediate-risk with high-dose daunorubicin.	Risk classification was based on the 2000 SWOG/ECOG classification.
7 + 3 Regimen	Pautas [45]/2010	Idarubicin: indicated for the treatment of AML in adults.	Multi-institutional, randomized, open-label trial	de novo AML	Induction daunorubicin 80 mg/m^2^ IV days 1–3 with Ara-C 200 mg/m^2^ IV continuous infusion days 1–7.	Induction Idarubicin 12 mg/m^2^ IV days 1–3 or 1–4 with Ara-C 200 mg/m^2^ continuous IV infusion days 1–7.	50–70 years	No risk groups excluded	CR rate 83% with idarubicin Days 1–3, 78% with idarubicin Days 1–4, and 70% with daunorubicin. No difference in OS, EFS or relapse incidence.	
7 + 3 +GO Regimen	Castaigne [46]/2012	Gemtuzumab ozogamicin: newly diagnosed AML, CD33+.	Multi-institutional, randomized, open-label trial	De novo AML, CD33+.	Induction daunorubicin and Ara-C with gemtuzumab ozogamicin 3 mg/m^2^ days 1, 4, 7. Similar regimen in consolidation.	Induction daunorubicin and Ara-C.	50–70 years	No risk groups excluded	Two-year HR of EFS was 0.56 (*p* < 0.01), and HR for OS was 0.58 (*p* < 0.05) for 7 + 3 + GO. Combined favorable and intermediate cytogenetic groups showed improved outcomes with gemtuzumab (HR 0.5, *p* = 0.08).	In follow-up study, 7 + 3 + GO improved EFS (HR: 0.66, *p* < 0.05) but not OS (0.81, *p* = 0.16) [27]. Risk classification was based on ISCN.
FLAG-Ida Regimen	Burnett [47]/2013	Fludarabine: NA	Multi-institutional, randomized, open-label trial	De novo or secondary AML.	Fludarabine 30 mg/m^2^ IV days 2–6, Ara-C 2 g/m^2^ days 2–6, G-CSF SC daily days 1–7, idarubicin 8 mg/m^2^ IV days 4–6.	Induction daunorubicin plus Ara-C with or without etoposide/gemtuzumab ozogamicin. Variables doses and schedules were used.	No age restriction	No risk groups excluded	CR rate 81% in ADE vs. 84% in FLAG-Ida (*p* = 0.2). No difference in 30- or 60-day mortality. Intermediate-risk cytogenetics had a lower relapse rate (OR 0.79, CI: 0.63–0.98) with FLAG-IDA.	Risk classification was based on MRC AML 10 Trial (15).
ADE Regimen	Willemze [48]/2013	Cytarabine Injection in combination with other approved drugs is indicated for remission induction in AML in adults.	Multi-institutional, randomized, open-label trial	De novo or secondary AML.	Daunorubicin 50 mg/m^2^ IV days 1, 3, 5 plus etoposide 50 mg/m^2^ days 1–5 plus Ara-C 3000 mg/m^2^ every 12 h IV infusion days 1, 3, 5, 7.	Daunorubicin 50 mg/m^2^ IV on days 1, 3, 5 plus etoposide 50 mg/m^2^ days 1–5 plus 10 days of Ara-C 100 mg/m^2^ as continuous IV infusion.	15–60 years	No risk groups excluded	6-year OS in high dose Ara-C, and the standard dose was 42.5% and 38.7% (*p* = 0.06). Subgroup analysis showed improved OS with high dose Ara-C in intermediate-risk (HR: 0.88, CI: 0.64–1.21).	In patients < 46 years, high-dose Ara-C was associated with improved 6-year OS (51.9% vs. 43.3%, *p* < 0.05). Intermediate-risk was defined as a normal karyotype or absence of low- and high-risk cytogenetics and of FLT3-ITD.
Azacitidine	Dombret [49]/2015	NA	Multi-institutional, randomized, open-label trial	De novo or secondary AML from MDS with >30% BM blasts who are not considered eligible for hematopoietic stem cell transplantation.	Azacitidine 75 mg/m^2^ SC daily for 7 consecutive days per 28-day treatment cycle	Investigators chose protocol-designated conventional care regimens (best supportive care, low-dose ara-c, or standard induction chemotherapy).	≥65 years	Intermediate- or poor-risk cytogenetics	Median OS 10.4 mos in azacitidine arm was vs. 6.5 mos in comparison arm (*p* = 0.1).	Outcomes with intermediate-risk cytogenetics were not statistically significant (HR: 0.9, *p* = 0.4). Risk classification was based on 2009 NCCN guidelines.
7 + 3 + Midostaurin Regimen	Stone [50]/2017	Midostaurin: newly diagnosed AML with *FLT3* mutation in combination with Ara-C and daunorubicin induction and Ara-C consolidation.	Multi-institutional, randomized, double-blind placebo-controlled trial	*FLT3*-ITD and TKD mutated. Not therapy-related.	Induction daunorubicin 60 mg/m^2^ IV days 1,2,3 with Ara-C 200 mg/m^2^ IV continuous infusion days 1–7 with midostaurin 50 mg orally twice daily, days 8–21.	Same but with placebo instead of midostaurin.	18–59 years	No risk groups excluded	HR for death in midostaurin group was 0.78 (*p* < 0.05). Subgroup analysis not statistically significant.	The trial was stratified to high (>0.7) vs. low (0.05–0.7) ITD or TKD allelic ratio.
**Newly Diagnosed AML-Induction-Ineligible**										
GO	Amadori [51]/2016	Gemtuzumab ozogamicin: newly diagnosed CD33-positive AML.	Multi-institutional, randomized, open-label trial	CD33+.	Gemtuzumab ozogamicin 6 mg/m^2^, Day 1, 3 mg/m^2^ Day?	Best supportive care.	>75 years or ≤75 years with WHO PS > 2	No risk groups excluded	HR for OS was 0.69 (*p* < 0.05). Subgroup analysis of combined favorable and intermediate cytogenetics showed improved outcomes with gemtuzumab (HR 0.52, *p* < 0.05).	Improvement in OS only seen with >80% CD33+ blasts.
Decitabine	Welch [52]/2016	NA	Single-institution, prospective, single-arm	Newly diagnosed or relapsed AML and MDS.	Decitabine 20 mg/m^2^ days 1–10 of 28-day cycles.	-	≥60 years	No risk groups excluded	ORR 46%. Median OS of favorable/intermediate-risk 10 mos.	Intermediate-risk cytogenetics in 5% of *TP53* mutated, 69% of *TP53* wild-type and 65% of *TP53* untested.
HMA + Sorafenib	Ohanian [53]/2018	Not approved.	Phase II, multi-institutional, open-label trial	Untreated patients with *FLT3* mutated AML unfit for standard chemotherapy.	Azacitidine 75 mg/m^2^ daily × 7 days and sorafenib 400 mg twice daily.	NA	≥60 years	No risk groups excluded	ORR 78%. Median OS 8.3 mos (range: 1–63).	63% of patients had a normal karyotype, 7% had a complex karyotype, and 15% had other karyotypic changes.
Low-dose Ara-C + Glasdegib	Cortes [54]/2019	Glasdegib: indicated in combination with low-dose Ara-C to treat newly diagnosed AML in adult patients ≥ 75 years old or with comorbidities that preclude the use of intensive induction chemotherapy.	Phase II, multi-institutional, randomized, open-label trial	Previously untreated AML or high-risk MDS unfit for intensive chemotherapy.	Glasdegib 100 mg orally QD continuously in 28-day cycles plus Ara-C 20 mg SC BID for 10 of 28 days.	Ara-C 20 mg SC BID for 10 per 28 days.	≥55 years	No risk groups excluded	Median OS 8.8 months in glasdegib group vs. 4.9 months in comparison group (*p* < 0.05).	Benefits mainly seen in good/intermediate groups combined (12.2 vs. 4.8 months, *p* < 0.05) but not in high-risk group (4.7 vs. 4.9, *p* = 0.06).
Enasidenib	Pollyea [55]/2019	Not FDA-approved.	Phase I, multi-institutional, open-label trial	Previously untreated *IDH2*-mutated AML unfit for standard AML treatments.	Enasedinib 100 mg orally once daily.	NA	≥18 years	No risk groups excluded	ORR 30.8%. Median OS 11.3 mos (CI: 5.7–15.1).	49% had intermediate-risk cytogenetics.
Azacitidne + Venetoclax	DiNardo [56]/2020	Venetoclax: it is approved in combination with azacitidine or decitabine, or low-dose cytarabine for the treatment of newly-diagnosed AML in adults 75 years or older or with comorbidities that preclude intensive induction chemotherapy. Accelerated approval.	Multi-institutional randomized, double-blind placebo-controlled trial	Ineligible for standard induction therapy due to coexisting conditions or age 75 years. Excluded patients with previous MPN or MDS treated with a hypomethylating agent.	Azacitidine 75 mg/m^2^ SC or IV days 1–7 every 28-days plus venetoclax with a target dose of 400 mg daily.	Azacitidine 75 mg/m^2^ SC or IV days 1–7 every 28-day cycle plus placebo.	≥18 years	Excluded patients with favorable-risk cytogenetics	Median OS in venetoclax group was 14.7 vs. 9.6 mos in the comparison (*p* < 0.05).	Statistically significant benefit in patients with intermediate-risk cytogenetics (HR 0.57, CI: 0.41–0.79) but not in high-risk group (HR: 0.78, CIL 0.54–1.12). Cytogenetics risk classification was based on the 2016 NCCN classification.
Ivosidenib	Roboz [57]/2020	Adult patients with newly-diagnosed AML ≥ 75 years old or with comorbidities that preclude intensive induction chemotherapy.	Phase I, multi-institutional, open-label trial	*IDH1*-mutated AML.	Ivosidenib 500 mg daily.	NA	≥18 years	No risk groups excluded	ORR 42.4%. Median OS 12.6 mos (CI: 4.5–25.7).	71% had intermediate-risk cytogenetics.
Ivosidenib + Azacitidine	Montesinos [58]	Adult patients with newly diagnosed *IDH1*-mutated uneligible for intensive treatment.	Phase 3, multi-institutional, double-blind, randomized trial	*IDH1*-mutated AML.	Ivosidenib (500 mg once daily) plus Azacitidine 75 mg/m^2^ daily × 28-day cycle.	Azacitidine 75 mg/m^2^ daily × 28-day cycle.	≥18 years	No risk groups excluded	Median OS was 24.0 months with experimental combination and 7.9 months with placebo and azacitidine (*p* = 0.001).	Similar toxicity profiles if expecting differentiation syndrome, higher in the experimental arm.

Abbreviations: CR = complete remission; ORR = overall response rate; EFS = event-free survival; OS = overall survival; HR = hazard ratio; mos = months; AML = acute myeloid leukemia; MDS = myelodysplastic syndromes; MRC-AML = acute myeloid leukemia with myelodysplasia-related changes; ITD = internal tandem duplication; TKD = tyrosine kinase domain; ISCN = International System for Human Cytogenetic Nomenclature; Ara-C = cytarabine; GO = gemtuzumab ozogamicin; FLAG = fludarabine, cytarabine, and filgrastim; Ida = idarubicin; ADE = cytarabine, daunorubicin hydrochloride, and etoposide phosphate; HMA = hypomethylating agents; SC = subcutaneously; BID = twice daily; NA = not applicable or not available.

**Table 4:** List of ongoing clinical trials including patients with newly diagnosed or relapsed/refractory intermediate-risk AML in the United States.

Clinical Trial Identifier	Name of Study	Design/Phase	Age Eligibility (Years)	Disease Characteristics	Study Start Date
NCT02152956	Flotetuzumab in Primary Induction Failure (PIF) or Early Relapse (ER) AML	Multicenter, phase ½, open-label	>18	R/R AML	June 2014
NCT02397720	Nivolumab and Azacitidine with or without Ipilimumab in Treating Patients with R/R or Newly Diagnosed AML	Phase 2, open-label study	>18	R/R AML	April 2015
NCT03190278	Study Evaluating Safety and Efficacy of UCART123 in Patients with R/R AML (AMELI-01)	Phase 1, open-label	18–65	R/R AML with >5% bone marrow blasts, CD123+	June 2017
NCT03067571	Daratumumab in Treating Patients with R/R AML or High-Risk MDS	Phase 2, open-label study	>18	R/R AML	October 2017
NCT03390296	OX40, Venetoclax, Avelumab, Glasdegib, Gemtuzumab Ozogamicin, and Azacitidine in Treating Patients with R/R AML	Phase 1b/2, open-label multi-arm study	>18	R/R AML	January 2018
NCT03504410	Study Evaluating Efficacy and Safety of CPI-613 in Combination with HD Cytarabine and Mitoxantrone Compared to HD Cytarabine and Mitoxantrone and Control Sub-groups: MEC and FLAG in Older Patients With R/R AML	Multicenter, phase 3, open-label, randomized study	>50	R/R AML	April 2018
NCT03672539	Liposome-encapsulated Daunorubicin-Cytarabine and Gemtuzumab Ozogamicin in Treating Patients with R/R AML or High-Risk MDS	Phase 2, open-label study	>18	CD33+ (≥3%), R/R AML	December 2018
NCT03839446	Phase II Study of the Combination of Mitoxantrone, Etoposide and Gemtuzumab Ozogamicin (MEGO) for Patients with AML refractory to Initial Standard Induction Therapy	Phase 2, open-label, single-arm study	18–75	R/R AML with CD33 expression in ≥30% of leukemic blasts on the bone marrow	February 2019
NCT03760523	Dose Escalation Study of Minnelide in R/R AML	Phase 1, dose-escalation study	>18	R/R AML ineligible for intensive chemotherapy	April 2019
NCT04219163	Chimeric Antigen Receptor T-cells for The Treatment of AML Expressing CLL-1 Antigen	Phase 1, open-label	≤75	R/R AML, at least 30% CLL-1+ blasts	July 2020
NCT04207190	Talazoparib and Gemtuzumab Ozogamicin for the Treatment of CD33 Positive R/R AML	Phase 1, open-label study	>18	CD33+ R/R AML with evidence of ≥5% myeloblasts in the bone marrow, peripheral blood, or in an extramedullary site by pathology	July 2020
NCT04278768	Dose Escalation/Expansion Trial of CA-4948 as Monotherapy and in Combination with Azacitidine or Venetoclax in Patients with AML or MDS	Phase 1/2, open-label	>18	AML (primary or secondary, including treatment-related) after failing at least 1 standard treatment	July 2020
NCT04435691	Magrolimab, Azacitidine, and Venetoclax for the Treatment of AML	Phase 1b/2, open-label study	>18	R/R AML	July 2020
NCT04659616	Pemigatinib after Chemotherapy for the Treatment of Newly Diagnosed AML	Multicenter, phase 1, open-label study	>18	Adverse- or intermediate-risk newly diagnosed AML	January 2021
NCT04666649	Pegcrisantaspase in Combination with Venetoclax for Treatment of R/R AML	Phase 1, open-label	>18	R/R AML	March 2021
NCT04669067	TL-895 and KRT-232 Study in AML	Multicenter, phase 1b/2, open-label	>18	*FLT3*-ITD or TKD mutation, *TP53* wild-type, R/R AML, at least one prior therapy, including a *FLT-3* inhibitor	March 2021
NCT04752163	DS-1594b with or without Azacitidine, Venetoclax, or Mini-HCVD for the Treatment of R/R AML or ALL	Phase 1b/2, open-label multi-arm study	>18	R/R AML or R/R ALL subjects with an MLLr or NPM1m	March 2021
NCT04582864	Flotetuzumab for relapsed AML and MDS Following Allo-HCT	Phase 2, open-label	>18	Relapsed AML	May 2021
NCT04789408	Study Evaluating the Safety of KITE-222 in Participants with R/R AML	Multicenter, phase 1, open-label	>18	R/R AML	July 2021
NCT05010122	ASTX727, Venetoclax, and Gilteritinib for the Treatment of Newly Diagnosed, R/R *FLT3*-Mutated AML or High-Risk MDS	Phase 1/2, open-label	>18	Newly diagnosed or R/R *FLT3*-mutated AML	July 2021
NCT04956042	Study of Fosciclopirox in Patients with R/R AML	Phase 1, open-label study	>18	R/R AML	August 2021
NCT03441048	Lintuzumab-Ac225 in Combination with Cladribine + Cytarabine + Filgastrim + Mitoxantrone (CLAG-M) for R/R AML	Single center, non-randomized, open-label phase 1	>18	R/R AML with >25% of blasts must be CD33 positive	May 2022

Abbreviations: R/R, relapsed or refractory; AML, acute myeloid leukemia; ALL, acute lymphoblastic leukemia; MDS, myelodysplastic syndrome; Allo-HCT, allogeneic hematopoietic cell transplantation; HD, high dose.
